# Are sunglasses appropriate for driving? Investigation and prototype for public testing

**DOI:** 10.1186/s12938-021-00881-9

**Published:** 2021-04-29

**Authors:** Artur D. Loureiro, Liliane Ventura

**Affiliations:** Electrical Engineering Department, São Carlos School of Engineering, University of São Paulo, Av. Trabalhador Sãocarlense 400, São Carlos, 13566-590 Brazil

**Keywords:** Sunglasses, Driving safety, Sunglasses category, *Q* factor, Traffic signals, ISO 12,312–1

## Abstract

**Background:**

Good vision through sunglasses is important to safety when driving and ISO 12312-1:2013 sets requirements for luminous transmittance and the transmittance of traffic signals.

**Methods:**

We measured the spectral transmittances, 380–780 nm in 5-nm steps, of 232 sunglasses lenses and calculated the luminous transmittance, category (1–4) and transmittance of red, yellow, green and blue traffic signals (*Q* values). Furthermore, we developed a prototype for the general public to self-check sunglasses regarding safety for driving. We combined a white LED, a photodetector, and calculations to measure luminous transmittance, traffic signal transmittance, category, and *Q*-factors in sunglasses.

**Results:**

Spectroscopy shows that 75% of sunglasses on the Brazilian market comply with ISO 12312-1:2013 requirements to be suitable for driving. The prototype was validated by testing 232 samples by trained users. Additionally, 60 other samples were tested by untrained users and results were compared to spectrophotometric measurements. Bland–Altman analyses showed no significant biases and 95% agreement of limits within the pre-defined tolerances for all measurements.

**Conclusions:**

Our prototype offers the general public a way to check whether their sunglasses are suitable for driving. As tested, 24.6% of sunglasses are not appropriate for driving and consumer must be more attentive to this information.

**Significance:**

Immediate attention regarding checking sunglasses for driving conditions is needed for non-certified sunglasses.

## Background

Our research group has carried out many studies related to sunglasses standards, including the adequacy of standard parameters to ensure eye safety in tropical countries, where solar irradiance is high, [1–3] as was done in the review on the former Brazilian ABNT NBR 15111 standard in 2013. This work is an extension of our research that emphasizes sunglasses and ocular health.

Previous research conducted by our group includes the development of a self-checking sunglasses kiosk, which informs the user about the lens category (0–4) and whether they have the UVA and UVB protection associated with their category; [4] and a national survey aimed at adapting the parameters of international sunglasses standards to the Brazilian behavioral and climatic situation, as well as studies reviewing requirements for international sunglasses standards. Thus, the present work is one of several systems that are being developed in our laboratory [4–8], to bridge the gap that exists between the public and sunglasses standards.

Our national survey started in 2012. The survey was conducted to characterize the typical profile of sunglasses use in Brazil. So far over 5000 people have been surveyed. This survey and permission for the public use of the responses have been approved by an Ethics Committee (trial registration approved by the Brazilian Ethics Committee, protocol number 160.248/2012—UFSCar). According to the respondents’ answers, 89.2% paid a range price for sunglasses of US $5–20 and 70.8% bought new sunglasses every 2–4 years. These data altered slightly over the years [3].

The public is usually interested in sunglasses style, eye comfort and UV protection when buying a pair of sunglasses [3]. Although these are relevant aspects, several others are noteworthy, for example safety for driving [9–13]. Lenses that are excessively dark could hinder object and traffic signal detections at safe distances, particularly in the shade. Moreover, lenses could excessively enhance or attenuate some colors, affecting signal detection. Thus, choosing inappropriate sunglasses may ultimately lead to dangerous situations [14, 15].

### Driving requirements from ISO 12312-1:2013

The international standard ISO 12312-1:2013 establishes requirements for all afocal sunglasses and clip-ons, intended for protection against solar radiation for general use, including road use and driving.

This standard grades sunglasses in five categories (0–4) according to visible transmittance (luminous transmittance) of their lenses, i.e., depending on the level of sun glare reduction of their lenses. Sunglasses are recommended for specific situations according to their category. The categories are rated between 0 (untinted lenses) and 4 (very dark lenses), the latter not appropriate for driving. Although not recommended for normal conditions, category 4 filters may be recommended in extreme high-luminance conditions, such as a desert or snowfields under full sunlight [16].

If lens luminous transmittance (380–780 nm) is less than 75%, sunglasses should not be used for road use and driving in twilight or at night. Those with transmittance less than 8% (category 4) are not appropriate for driving at any time. Additionally, regarding road use and safe driving conditions, the spectral transmittance of filters for the 475–650 nm range should not be less than 0.2 times the luminous transmittance. Sunglasses should also fit requirements for color detection that are presented later (*Q* factor limits) [16].

Table 1 reproduces the limits of luminous transmittance for establishing the categories of sunglasses according to ISO 12312-1:2013.

**Table 1:** Sunglasses category limits, adapted from ISO 12312-1:2013

Filter category	Visible spectral range
	Range of luminous transmittance $\tau_{\mathsf{v}}$ 380–780 nm
0	$\tau_{\mathsf{v}}$ > 80%
1	43% < $\tau_{\mathsf{v}}$ ≤ 80%
2	18% < $\tau_{\mathsf{v}}$ ≤ 43%
3	8% < $\tau_{\mathsf{v}}$ ≤ 18%
4	3% < $\tau_{\mathsf{v}}$ ≤ 8%

Overlapping of the luminous transmittance values should not exceed 2% (absolute) between categories 0, 1, 2 and 3 and overlapping between categories 3 and 4 is not allowed.

The luminous transmittance, $\tau_{\mathsf{v}}$, is defined as

1 $$\tau_{\mathsf{v}} = \frac{{\int\limits_{380}^{780} {\tau \left( \lambda \right)V\left( \lambda \right)S_{\mathsf{D65}} \left( \lambda \right)d\lambda } }}{{\int\limits_{380}^{780} {V\left( \lambda \right)S_{\mathsf{D65}} \left( \lambda \right)d\lambda } }} = \frac{{\int\limits_{380}^{780} {\tau \left( \lambda \right)W_{\mathsf{v}} \left( \lambda \right)d\lambda } }}{{\int\limits_{380}^{780} {W_{\mathsf{v}} \left( \lambda \right)d\lambda } }},$$

in which $\tau \left( \lambda \right)$ is the spectral transmittance of the filter, $V\left( \lambda \right)$ is the spectral luminous efficiency function for photopic vision, $S_{{{\mathsf{D65}}}} \left( \lambda \right)$ is the visible part of the solar spectrum at sea level for air mass 2 (terrestrial solar spectrum occurring when the sun’s position vector is 60 degrees from the zenith) [16]. For the sake of convenience, we defined $W_{\mathsf{v}} \left( \lambda \right)$ as the luminous weighting function, $V\left( \lambda \right)S_{{{\mathsf{D65}}}} \left( \lambda \right)$.

Each category has a recommended use. Darker lenses (less luminous transmittance) are recommended for environments with higher solar incidence, while lenses with luminous transmittance of less than 3% are not considered appropriate to be used.

Traffic signal transmittances, $\tau_{{{\mathsf{signal}}}}$, are defined for red, yellow, green and blue as

2 $$\tau_{{{\mathsf{signal}}}} = \frac{{\int\limits_{380}^{780} {\tau \left( \lambda \right)V\left( \lambda \right)E_{{{\mathsf{signal}}}} \left( \lambda \right)d\lambda } }}{{\int\limits_{380}^{780} {V\left( \lambda \right)E_{{{\mathsf{signal}}}} \left( \lambda \right)d\lambda } }} = \frac{{\int\limits_{380}^{780} {\tau \left( \lambda \right)W_{{{\mathsf{signal}}}} \left( \lambda \right)d\lambda } }}{{\int\limits_{380}^{780} {W_{{{\mathsf{signal}}}} \left( \lambda \right)d\lambda } }},$$

where $E_{{{\mathsf{signal}}}} \left( \lambda \right)$ is the spectral energy distribution of red, yellow, green, or blue traffic signals, which is different for traffic lights lit by incandescent and LED lamps and is available in the standards [16]. For the sake of convenience, we defined $W_{{{\mathsf{signal}}}} \left( \lambda \right)$ as the spectral weighting functions for traffic lights, $V\left( \lambda \right)E_{{{\mathsf{signal}}}} \left( \lambda \right)$.

There is also a relative visual attenuation quotient for traffic luminous detection, denoted by $Q_{{{\mathsf{signal}}}}$, and defined as

3 $$Q_{{{\mathsf{signal}}}} = \frac{{\tau_{{{\mathsf{signal}}}} }}{{\tau_{\mathsf{v}} }}.$$

As previously stated, traffic signal transmittances and consequently, relative visual attenuation quotients are defined for incandescent and LED lights; however, standard requirements take into account only the relative visual attenuation quotients (*Q* factors) related to incandescent lights. Data related to LED lights are for information only.

According to the standards, to be suitable for driving, sunglasses should belong to categories 0, 1, 2 or 3 ($\tau_{\mathsf{v}} >$ 8%), and *Q* factors should not be less than 0.80 for the red signal light, and not less than 0.60 for the yellow, green and blue signal lights. If any requirement for driving is not met, the manufacturer should include a warning stating that sunglasses are not suitable for driving.

It is important for a community to be able to test their sunglasses to assess their optical properties, besides UV protection, such as the categories and if they are suitable for driving land vehicles. Since transmittance tests are complex and require using scientific equipment, the public does not have any access to testing their own sunglasses. Hence, we have developed a prototype for the public for self-testing their sunglasses, to check their category and if they are suitable for driving.

The prototype has been developed by the ISO 12312-1:2013 requirements, as well as a friendly interface for the public, reporting the luminous transmittance, the category of sunglasses, and whether sunglasses are appropriate for driving (*Q* factors for red, yellow and green signals). The prototype is not intended to be used for checking compliances with ISO 12312-1:2013, but to advise the general public on appropriate sunglasses for safe driving, for countries where standards are not compulsory, as in Brazil.

In this work we have tested sunglasses, which are addressed as unbranded and labeled samples. The unbranded (unlabeled) samples were purchased on the streets as: 10% are samples from Europe (Spain, Portugal and Germany); 15% are from the United States; 20% are from Brazil (north, northeast, south and southeast parts of the country). Remaining 55% are samples from China, which are sold on the Brazilian market. The relation of unbranded (222) and tagged sunglasses (10) is representative of the sunglasses market in Brazil, as shown in a national survey conducted by our team [3].

## Results

### Spectrophotometric analysis of sunglasses on the Brazilian market

We tested 232 samples (222 from unbranded sunglasses and 10 from tagged ones). Most samples belong to category 3 (42.2%), 32.8% to category 2, 19% to category 4 and 3% to category 1, which are the lightest tinted lenses. In the set, there was no category 0 lens (clear lens), and for 3% (seven samples), the luminous transmittance was less than 3%, thus they are not compliant to ISO 12312-1:2013 at all and are not categorized (not appropriate for driving whatsoever).

**Fig. 1:**
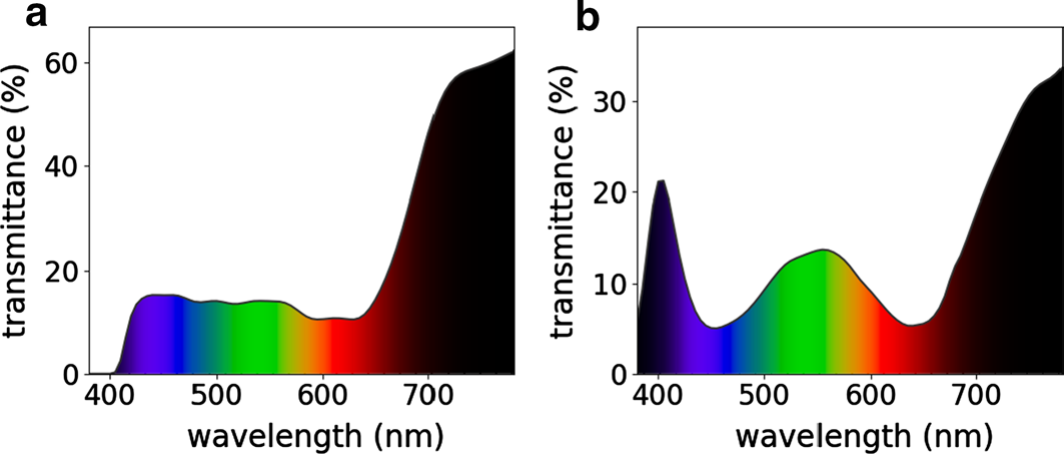
Transmittance spectrum (380–780 nm) of sample code: **a** LE 0 381, which meets driving requirements from ISO 12312-1:2013; **b** LE 23 4, which does not meet these requirements, for the red region, *Q* factor = 0.66

Figure 1a shows the transmittance spectrum of a pair of sunglasses which meet driving requirements from ISO 12312-1:2013, and Fig. 3b, shows the transmittance spectrum of a pair of sunglasses that does not meet these requirements, for the red region, *Q* factor = 0.66.

We measured 232 samples using a spectrophotometer. From these, 175 are suitable for driving and 57 are not suitable for driving (06 samples for failing the condition of $Q_{{{\mathsf{red}}}}$ > 0.8; 44 for belonging to category 4; and seven samples for failing the condition of $\tau_{\mathsf{v}} > 3\%$). In two pairs of sunglasses, the lenses belonging to each pair (right and left lenses) have different categories.

### Prototype validation

The prototype’s measurements matched the results from spectroscopy related to the driving suitability of the 232 samples.

**Fig. 2:**
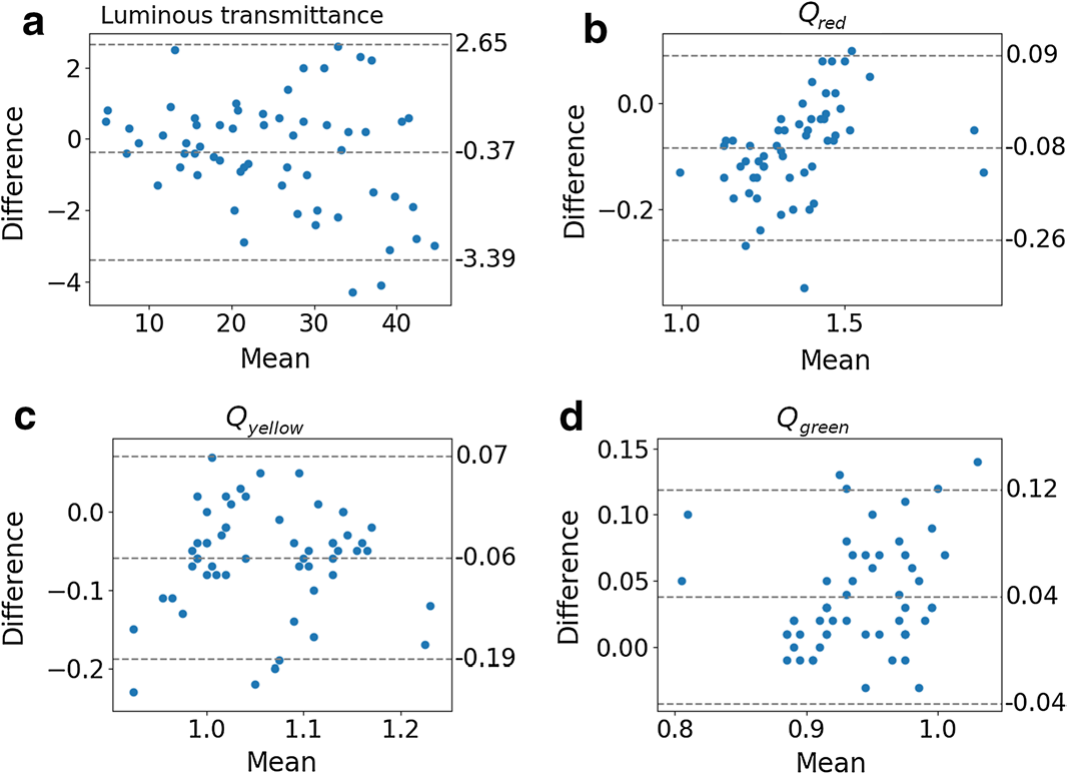
Bland–Altman plots for: **a** luminous transmittance; and traffic signal *Q* factors—**b** red; **c** yellow; **d** green

Of the 60 samples tested, one belonging to category 1 ($\tau_{\mathsf{v}}$ = 43.3%) was reported as belonging to category 2 (41.0%). For all 59 remaining lenses, the category and suitability for direction measurements were in line with the measurements made using a spectrophotometer. Furthermore, out of the 60 selected lenses, four samples presented luminous transmittance less than 8%, and were excluded from further traffic signal measurements. Therefore, the luminous transmittances were determined for all 60 lenses and the traffic light visual attenuation quotients were determined for the remaining 56 lenses. Bland–Altman plots are shown in Fig. 2.

The bias and 95%-limit-of-agreement interval are shown in Table 2. These values are within pre-defined tolerances.

**Table 2:** The bias and the 95%-limit-of-agreement interval for each measure

	Bias	95%-limit-of-agreement Interval
$\tau_{\mathsf{v}}$	− 0.37	[− 3.39, 2.65]
$Q_{{{\mathsf{red}}}}$	− 0.08	[− 0.26, 0.09]
$Q_{{{\mathsf{yellow}}}}$	− 0.06	[− 0.19, 0.07]
$Q_{{{\mathsf{green}}}}$	0.04	[− 0.04, 0.12]

Bland–Altman plots (Fig. 2) indicated consistent variability across the graphs, without trends, for all plots. On average, prototype measures were lower than gold standard ones except for $Q_{{{\mathsf{green}}}}$, which means that a possible correction of the obtained values using an offset, a proportional gain or otherwise, should be done in such a way as to increase the obtained values, except for $Q_{{{\mathsf{green}}}}$, which should be reduced.

## Discussion

Bland–Altman plots presented non-significant biases and narrow 95% limits of agreement within pre-defined tolerances, for all plots. The plots also indicated consistent variability across the graphs, without trends, for all plots. Therefore, prototype measurements were accurate compared to spectrophotometer gold standard measurements within pre-defined tolerances.

Although one of the lenses diverged in the category measurement, for that particular lens, luminous transmittance was in the range of the category overlap, bearing the limit of the range (2.3%).

It is worth highlighting that ISO certification is not compulsory in Brazil. Therefore, sunglasses that are unsuitable for driving might not have any warning.

## Conclusions

### Sunglasses on the Brazilian market

This paper evaluated a set of sunglasses, which represents the Brazilian market to check if the products that are used by consumers are appropriate for driving land vehicles safely.

Most of them, i.e., 75% meet the necessary requirements for driving according to ISO 12312-1:2013. Only 3% of them are not compliant with ISO 12312-1:2013 at all, since their lenses are extremely dark.

### Prototype

The developed prototype cannot be used to check sunglasses in compliance with the ISO 12312-1:2013 standard as to suitability of driving. However, our results show that the system we propose is worth being used by the general public to access information from their own sunglasses, as well as to educate themselves about the driving requirements, which must be considered when buying a pair of sunglasses.

The intelligibility of the tests, with their self-explanatory screens, makes the device interesting to sell sunglasses. At the same time it adds value to sales and acts as additional advertising, the device can be used to educate salespeople, who normally do not have access to information about the suitability for driving.

It is important that the population has access to information about the suitability for driving using sunglasses and a way to test them before purchasing. Just as the market has been successful in advertising over the years concerning UV protection in sunglasses, so should suitability for driving.

## Methods

### Spectroscopy measurements

Electromagnetic spectroscopy—transmittance—was performed on sunglasses with the CARY 5000 (VARIAN) spectrophotometer, which is a double-beam system, in the visible range, from 380 to 780 nm, with 5-nm steps.

ISO 12312-1:2013 establishes that spectroscopic measurements should be performed so that the optical path from the light source to the sensor passes through the geometric center of the lens, in a region of 5 mm in diameter.

The transmittance spectrum of the sunglasses’ lenses in the visible range (luminous transmittance), as well as traffic signal color ranges, led to determining the categories of the lenses, as well as the *Q* factors for red, yellow and green. From these values, we check whether the sunglasses comply with item 5.3.2 Requirements for road use and driving.

### Prototype development

This study has been submitted to the Ethical Committee—CONEP (Conselho Nacional de Ética em Pesquisa—National Consul of Ethics in Research) and it has been approved under the Registration number: 160.248—CCAE: 02140312.5.0000.5504 at the Ethical Committee of CEP UFSCar. The study is being conducted in accordance with the provisions of the Declaration of Helsinki for experimentation involving human ethics.

The developed system consists of a high brightness white LED (OSRAM Golden Dragon Ultra White LED, whose correlated color temperature is 6500 K—manufacturer’s claim) and a four-channel photodetector (AMS TAOS TCS3472). The four-channel photodetector performs four measurements simultaneously using four different weighting functions as if it were four independent sensors.

Before sample measurements, the system measures four baselines, denoted as CALIBRATION of the system. The transmittance measurements are performed with weights defined by hardware characteristics (spectral emission from white LED and spectral response from each TCS3472 channel).

Photodetectors provide integrated responses with specific spectral weights and, in this particular model, different spectral weights for each one of its four channels. The baseline consists of illuminating the photodetector directly by the light source with an open optical path and registering this value as the 100% transmittance. Subsequently, sunglasses lenses are positioned in the optical path, and the response of the photodetector is measured.

For linear response photodetectors, the ratio of the measured value of the sample and baseline value is equal to the light transmittance weighted with a spectral function given by the term-to-term product of the spectral emission of the light source and the spectral response of the photodetector. If a photodetector has no linear response, a simple mathematical correction should be made.

**Fig. 3:**
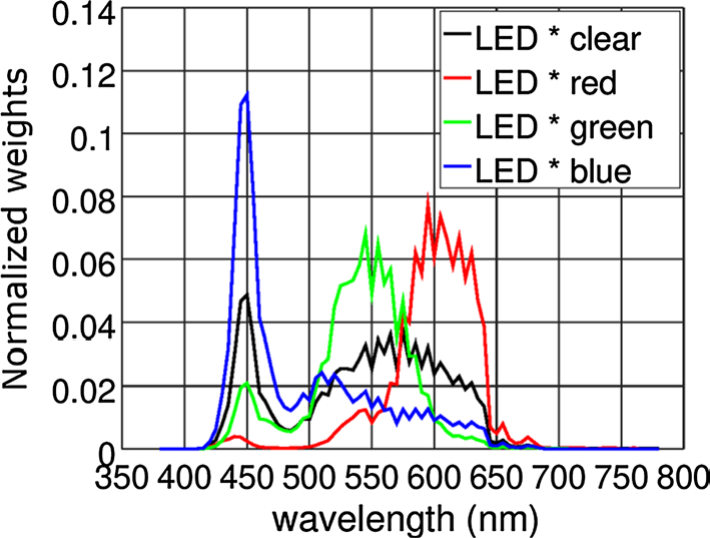
The four spectral weighting functions generated by TCS3472 illuminated by Golden Dragon LED. These functions, that are not similar to the ones defined in ISO 12312-1:2013, were linearly combined to approximate the standard’s weighting functions

The TCS3472 photodetector provides four different responses. This photodetector consists of a 3 × 4 photodiode array, which is composed of red-filtered, green-filtered, blue-filtered, and clear (unfiltered) photodiodes. Therefore, the transmittance of the sample can be measured with four different known weighting functions (Fig. 3). Furthermore, the four known weighting functions are linearly combined to estimate the desired weighting functions, in this case the desired weighting functions are the ones for luminous and traffic signals transmittances defined in ISO 12312-1:2013. In other words, we obtained four weighting functions (Fig. 3) that are not similar to the standard and we used these functions to approximate the standard’s ones.

**Fig. 4:**
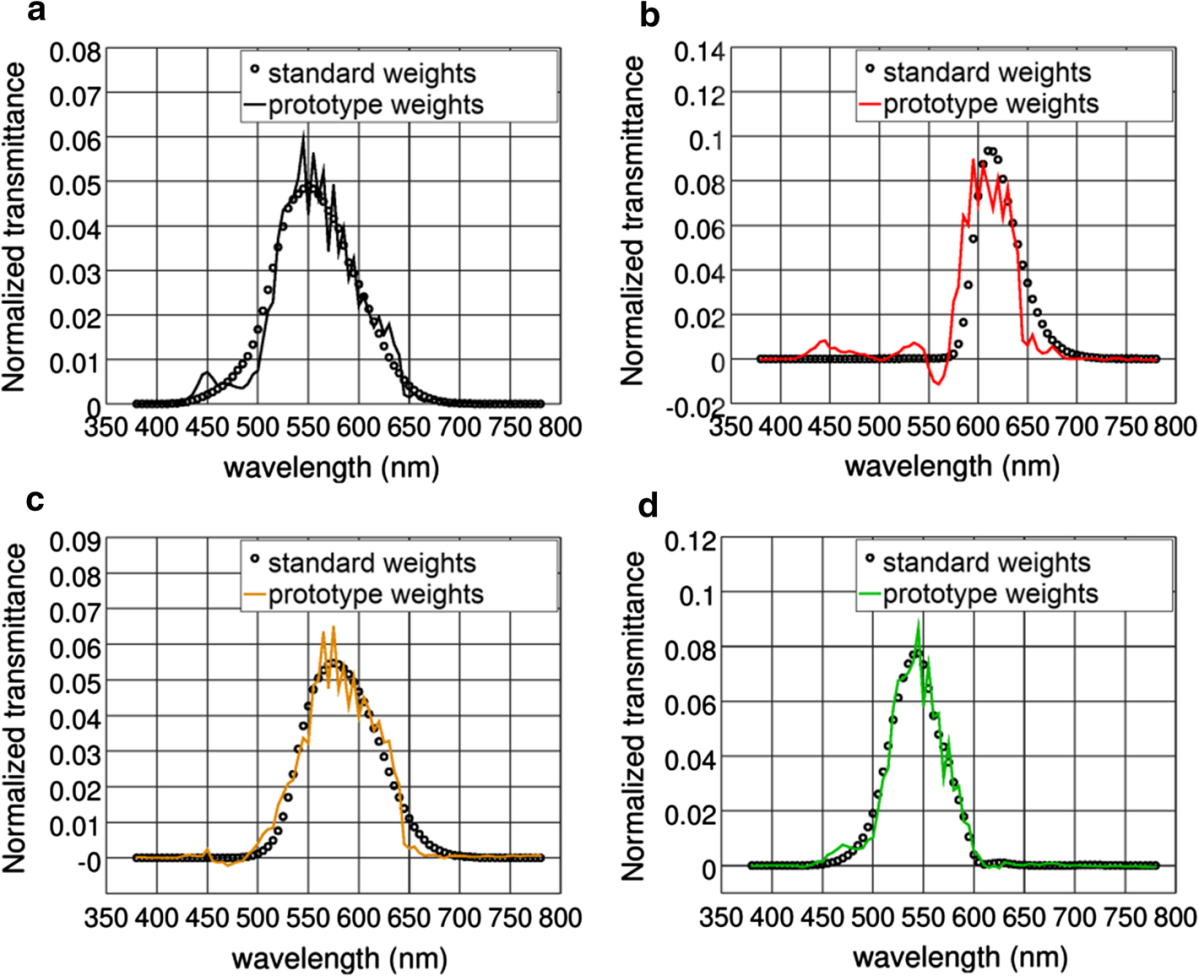
The prototype’s weighting functions (continuous line), obtained by linear combination of the functions from Fig. 3, and the weighting functions established by ISO 12312-1:2013 (empty circles) for the following transmittances **a** luminous; **b** red; **c** yellow; **d** green

The approximated weighting functions used to measure luminous and traffic signals (red, yellow and green) transmittances are shown in Fig. 4 superimposed with the standard's functions they approximate.

We developed a program in C++ language to measure transmittance values using a white LED and a TCS3472 sensor (weights shown in Fig. 3). From the measured values, it calculates transmittance values weighted by prototype weights shown in Fig. 4. Finally, it reports whether tested sunglasses are suitable for driving according to category and *Q* factor requirements from ISO 12312-1:2013. It is worth mentioning that our prototype measures are not intended to be used in certifications, but to provide information about sunglasses to the general public.

Touchscreen display DWIN 4.3ʺ DMT48270T043_18WT was used as the user interface.

**Fig. 5:**
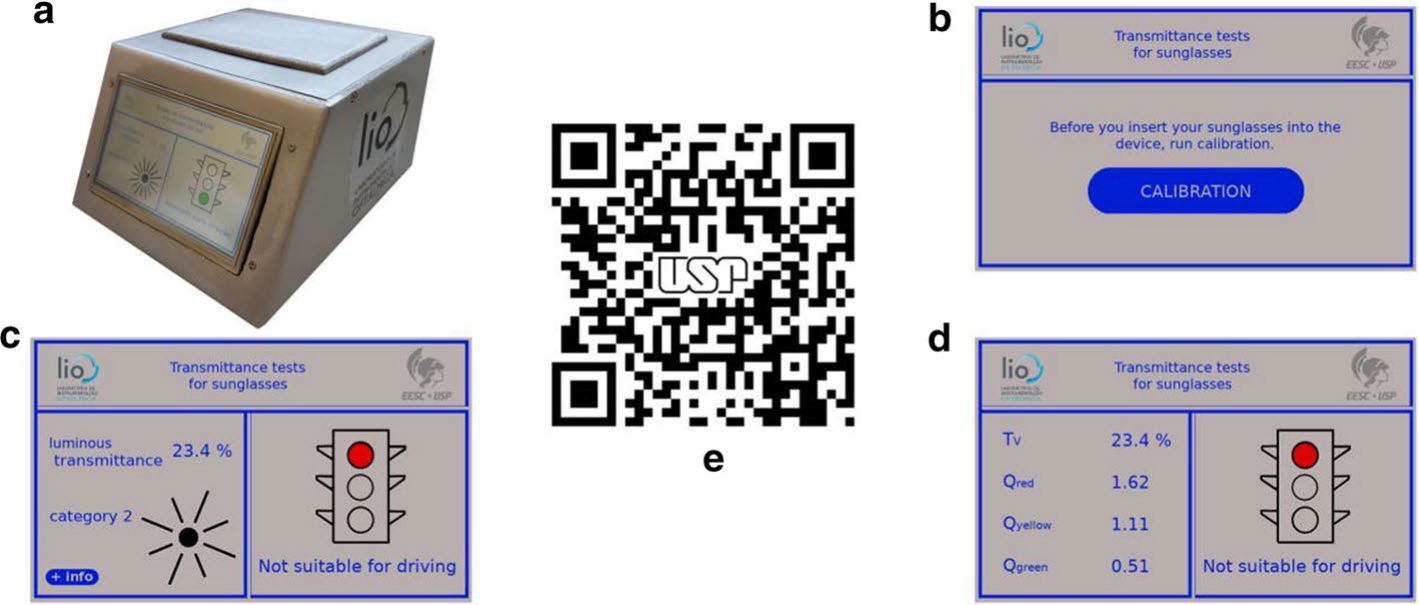
**a** Prototype with touch screen display; **b** default initial screen; **c** primary result screen of tested sunglasses; **d** secondary result screen with details; **e** QR code of prototype video

Figure 5 shows the prototype (Fig. 5a) and three of its screens, as well as a QR code that accesses a video of the prototype.

The prototype’s home screen invites users to test their sunglasses and press the CALIBRATION button (Fig. 5b). After pressing the CALIBRATION button, the device measures the baseline and then prompts the user to place their sunglasses inside the device. After positioning the sunglasses, the user presses the TEST button and the device shows the results screen (Fig. 5c), showing whether the tested sunglasses are suitable for driving, their luminous transmittance, their category and their recommended use.

To access the user’s sunglasses traffic signal visual attenuation quotients (Fig. 5d), the user should tap on the MORE INFORMATION button.

### Measurement procedures

We tested 232 samples using the VARIAN CARY 5000 spectrophotometer. The spectrophotometer was previously calibrated with holmium oxide in 1.4 M perchloric acid solution, and peaks were checked at 241.15 nm, 287.15 nm, 361.5 nm, 486.0 nm and 536.3 nm. There was a 0.05 nm shift.

At first, during the development of the prototype, to ensure that the results of the prototype would be reliable, the same 232 samples were tested on the prototype by a transmittance measurement specialist.

Additionally, the sunglasses were tested on the prototype, by non-trained users, who were people on the street of São Carlos (SP) in Brazil, as well as on the University of São Paulo campus in the same town, out of which 60 samples were randomly selected and submitted to transmittance spectroscopy in the VARIAN CARY 5000 spectrophotometer. This evaluation reflects the validity of the prototype results by the non-trained public.

### Prototype validation

We analyzed the agreement between our system and gold standard spectrophotometer by using the Bland–Altman method. For luminous transmittance, the bias was adopted as significant if it was greater than 0.5% (absolute). The 95% interval of agreement was considered wide if the upper limit is greater than 5% (absolute) or the lower limit is lesser than -5% (absolute). For the *Q* factors, the bias was adopted as significant if it was greater than 0.1. The 95% interval of agreement was considered wide if the upper limit was greater than 0.3 or the lower limit was less than -0.3.

## Data Availability

Not applicable.

## Abbreviations

$$\tau_{\mathsf{v}}$$: Luminous transmittance
LED: Light-emitting diode
$$Q_{\mathsf{red}}$$: Red *Q* factor
$$Q_{\mathsf{yellow}}$$: Yellow *Q* factor
$$Q_{\mathsf{green}}$$: Green *Q* factor
$$Q_{\mathsf{blue}}$$: Blue *Q* factor
UV: Ultraviolet
